# Low genetic variation and strong genetic structure across a range of geographical scales in European smelt ( *Osmerus eperlanus* L.)

**DOI:** 10.1111/jfb.70093

**Published:** 2025-05-29

**Authors:** Niall J. McKeown, Alix N. Taylor, Niklas Tysklind, Amy J.E. Healey, Martin I. Taylor, Andy R. Beaumont, Ian D. McCarthy

**Affiliations:** ^1^ Department of Life Sciences Edward Llwyd Building, Aberystwyth University Ceredigion UK; ^2^ School of Ocean Sciences Bangor University Anglesey UK; ^3^ INRAE, UMR EcoFoG (Agroparistech, CNRS, Cirad Université des Antilles, Université de la Guyane) Campus Agronomique Kourou France; ^4^ School of Biological Sciences University of East Anglia, Norwich Research Park Norwich UK

**Keywords:** European smelt, genetic variation, *Osmerus eperlanus*, phylogeographic history, population structure, small pelagic

## Abstract

Populations of anadromous European smelt (*Osmerus eperlanus* L.) are declining across its range with mitigation efforts for this ecologically important species hindered by a lack of demographic information. Here, mitochondrial DNA (mtDNA) and microsatellite analyses were used to describe historical and recurrent demographics for the species across a large part of its range. mtDNA revealed a shallow phylogeographic structure indicating a cohesive ancestral population, low overall haplotype and nucleotide diversities. However, microsatellites revealed unexpectedly high genetic structuring (*F*_ST_ = 0.15; *p* < 0.0001), including (i) isolation by distance effects over various scales, (ii) separation between Baltic and Atlantic samples and (iii) the highest interpopulation divergence and the lowest intrapopulation variation among UK sites. The results indicate that despite considerable dispersal potential, there is strong structuring among rivers, which should be recognised as separate management units. Furthermore, individual clustering analyses revealed further population separation within waterways and the need to resolve isolating mechanisms. Overall levels of genetic variation were found to be lower than those reported for other osmerids, with evidence suggesting that a considerable portion of ancestral variation has been eroded. As such, low genetic variation may limit resilience to environmental change. Proactive management strategies are discussed, with the prioritisation of UK populations recommended.

## 1 INTRODUCTION

Knowledge of population genetic structure and connectivity among populations within species is essential for designing and assessing effective strategies for biodiversity conservation and resource management (Frankham, 2005). For anadromous species (i.e., those that ascend rivers from marine waters as adults to spawn), marine migrations may create opportunities for extensive dispersal and gene flow, whereas natal homing/philopatry may restrict connectivity among spawning groups and foster local adaptation (Quinn et al., 2001). The dendritic nature of freshwater habitats, as well as anthropogenic activities driving fragmentation, may be important extrinsic factors impacting population demographics (Dynesius & Nilsson, 1994). The recurrent interplay between migration dynamics and the aquatic landscape can influence population growth by immigration, extinction risk and recolonisation of drainages, as well as the maintenance of phenotypic, adaptive and genetic diversity (Hasselman et al., 2013). Studies that span large parts of a species' range may also reveal signatures of historical contingency. In formerly glaciated areas, these may include distinct lineages, endemic genetic variants and the disproportionate partitioning of genetic variation in former refugial areas that may warrant conservation prioritisation (Hasselman et al., 2013).

The European smelt, *Osmerus eperlanus* L., occurs as an anadromous form in coastal and estuarine waters of Europe, historically ranging from the west coast of France to the Baltic, Barents and White Seas in the north, as well as the southern North Sea, British Isles and Ireland (Froese & Pauly, 2023). Relict landlocked lacustrine populations occur in Scandinavia, Baltic and White/Barents Sea regions (Froese & Pauly, 2023). Some populations have also become landlocked in southern waters owing to anthropogenic habitat alterations (Tulp et al., 2013). Smelt play an important role in food webs as predators of zooplankton and prey for larger piscivorous fish and birds in brackish and freshwater ecosystems (Hammar et al., 2018; Sandlund et al., 2004; Taal et al., 2014; Thiel et al., 1996). Sandlund et al. (2004) likened it to a ‘keystone species’ on account of its fundamental importance within ecosystems, whereas Svardson (1976) used the term ‘buffer species’ to describe its role in reducing negative interactions between species such as whitefish *Coregonus* spp. and Arctic charr *Salvelinus alpinus* in some lakes. Adult anadromous smelt migrate into the upper reaches of estuaries and parts of rivers above the head of the tide to spawn in spring, between February and April depending on population. The young are often found between the upper and lower tidal limits within estuaries, suggesting that larvae are dispersed from the initial spawning sites (Quigley et al., 2004). Juveniles maintain their position in the brackish water of the upper estuary feeding on macroinvertebrates as they grow before moving out to sea to feed and become mature at 2–3 years of age. Although movements between lagoons and coastal waters have been shown (Fernández‐Alías et al., 2022), it is not known how far the adults roam while in the sea, with studies suggesting limited coastal movements and/or regional variability in adult dispersal (Elliott et al., 2023; Tulp et al., 2013), nor is it known to what extent they are philopatric, returning to the river in which they hatched to spawn. During the spawning season, within‐river migrations of up to 25 km and freshwater residency times of up to 64 days have been reported (Colclough & Coates, 2013; Moore et al., 2016).

Smelt have decreased considerably in areas across its historical distribution (Wilson & Veneranta, 2019). Although previously reported as far south as the Gironde estuary in France, this population is believed extirpated (Pronier & Rochard, 1998), and the southern limit may be the Loire in Brittany (GIP Loire Estuaire, 2023). In the UK, smelt populations are now absent from approximately 33% of the estuaries and rivers where they historically occurred in England and Wales (Maitland, 2003) and 80% in Scotland (Maitland & Lyle, 1996). The only fully landlocked population in Britain, in Rostherne Mere, went extinct in the 1920s (Maitland, 2003). In Ireland, there are three known anadromous populations and no extant landlocked populations (Quigley, 1996; Quigley et al., 2004). The species still supports economically and culturally significant commercial and recreational fisheries in some areas, most notably Baltic countries; however, such fisheries have also reported considerable recent declines (Sendek & Bogdanov, 2019; Svanberg et al., 2019). The observed population declines are linked to factors such as overfishing, including illegal, unreported and unregulated fishing; pollution; destruction of spawning/nursery habitats; and the physical obstruction of spawning migrations by the erection of dams and weirs (Maitland, 2007; Sendek & Bogdanov, 2019; Wilson & Veneranta, 2019).

Currently, there is no cohesive international conservation management strategy for the species (Wilson & Veneranta, 2019). This can, in a large part, be attributed to a general lack of both local and integrated cross‐border population demographic information. The International Council for the Exploration of the Sea (ICES) recognises the species as ‘data deficient’, whereas the International Union for Conservation of Nature (IUCN), which currently lists the species in the ‘least concern’ category, states that the species' population trend is ‘unknown’ (Freyhof, 2011). There is a fundamental lack of knowledge about the patterns and processes of population connectivity. It is uncertain as to how far adults may disperse at sea (although this may be limited to close inshore; Elliott et al., 2023), the occurrence and/or accuracy of natal homing, as well as potential for larval dispersal. Population genetic studies have reported considerable regional variability in population genetic structuring across US and Canadian waters in the congeneric *Osmerus mordax*, pointing to complex roles of seascape structuring, larval and adult behaviours and local adaptation. Since the development of microsatellite markers for European smelt by Taylor et al. (2011), the only population genetic studies of *O. eperlanus* to date were restricted to Scandinavian landlocked populations (Hagenlund et al., 2015) and the Curonian Lagoon and nearby waters in the Baltic Sea (Fernández‐Alías et al., 2022). Therefore, the purpose of our study was to investigate the population genetic structure of anadromous smelt populations across a large part of the species' range. As the geographical area under study has been profoundly affected by the Pleistocene glaciations, mitochondrial DNA (mtDNA) and microsatellite variation were assessed to help disentangle historical and recurrent demographic factors, as recommended by Bradbury et al. (2011). From the resolved spatial structure, the specific objectives were to (i) describe the species' phylogeographic history; (ii) identify contemporary patterns and drivers of connectivity; (iii) assess levels of genetic variation among anadromous and landlocked populations and at the species level in comparison to other osmerids; and (iv) interpret patterns in the context of proactive management strategies at population and species levels.

## 2 MATERIALS AND METHODS

### 2.1 Sample collection and genotyping

**FIGURE 1:**
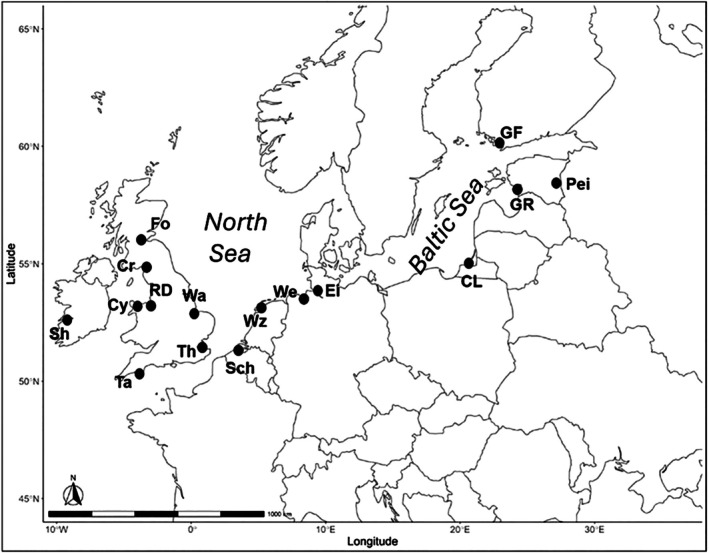
Approximate locations of sample sites of European smelt *Osmerus eperlanus* included in this study. Samples codes are as follows: Sh = Shannon, Cr = Cree, Cy = Conwy, RD = River Dee, Ta = Tamar, Th = Thames, Wa = the Wash, Fo = Forth, Sch = Scheldt, Wz = Waddenzee, We = Weser, Cl = Curonian Lagoon, GR = Gulf of Riga, GF = Gulf of Finland, Pei = Lake Peipsi. Microsatellite and mitochondrial DNA (mtDNA) genotypes were collected from all sites except Sh, Cy, RD and GF (mtDNA only) and GR and Pei (microsatellite only).

Samples in the form of muscle or scales were obtained from 16 sites across Europe (Figure 1; Table S1) between 2004 and 2008 (with the exception of samples from the Tamar collected in the 1990s). Most samples were obtained through scientific netting surveys, although some samples were obtained from commercial anglers (Thames, The Wash, Gulf of Finland) or from fish screens at power stations (Forth). The majority of smelt sampled were from anadromous populations with the exception of Lake Peipsi (landlocked) in Estonia (Figure 1). All samples were genotyped for a combination of mtDNA (Table 1) and microsatellite loci (Table 2) except for the Shannon, Conwy, Dee and Gulf of Finland samples (mtDNA only) and Gulf of Riga and Lake Peipsi samples (microsatellites only). DNA was extracted using the phenol‐chloroform method from Winnepenninckx et al. (1993). A 788‐bp fragment of the mtDNA cytochrome B gene was polymerase chain reaction (PCR)‐amplified using primers (F 5′‐AAACTGCCACAGCATTTTCC‐3′ and R 5′‐GAAGGGGCGGAAAGTTAGTC‐3′) designed from sequences on GenBank. mtDNA PCRs were performed in 10‐μL volumes using a thermoprofile comprising 95°C for 3 min followed by 35 cycles of 95°C by 30s, 55°C for 30s, 72°C for 30s, with a final extension of 72°C for 180 s. Amplicons were purified and sequenced using Big Dye technology and an ABI 3500 DNA analyser. Microsatellite genotyping was performed using the primers and conditions for 12 loci (Oep1.35, Oep2.3, Oep3.8, Oep5.38, Oep5.39, Oep5.59, Oep5.67, Oep6.1, Oep6.42, Oep7.11, Oep7.5, Osmolav‐12) described by Taylor et al. (2011).

**TABLE 1:** Overview of mitochondrial DNA (mtDNA) variation in European smelt *Osmerus eperlanus*, including haplotype (*H*) and nucleotide (π) diversity.

	Nhap	H1	Private haplotypes	Non‐private haplotypes	*H*	π
Shannon	3	14		H10 (2), H17 (1)	0.324	0.0005
Cree	2	10	H2 (6)		0.5	0.0007
Conwy	1	7			0	0
Dee	1	8			0	0
Tamar	1	8			0	0
Thames	4	7	H24 (1)	H22 (2), H23 (6), H24 (1)	0.692	0.0029
Wash	4	7	H28 (1)	H22 (3), H23 (5)	0.717	0.0028
Forth	1	14			0	0
Scheldt	4	10	H21 (1)	H10 (1), H17 (1)	0.423	0.0009
Waddenzee	5	11	H25 (1), H26 (1), H27 (1)	H17 (2)	0.533	0.0012
Weser	6	10	H18 (1), H19 (1), H20 (1)	H10 (1), H17 (2)	0.617	0.0011
Eider	6	11	H3 (1), H4 (1), H5 (1), H6 (1), H7 (1)		0.542	0.0011
Curonian Lagoon	6	10	H13 (1), H14 (1), H15 (1), H16 (1)	H9 (2)	0.518	0.0015
Gulf of Finland	6	11	H8 (1), H11 (1), H12 (1)	H9 (1), H10 (1)	0.542	0.0017

*Note:* Nhap reports the number of different haplotypes within a site. H1 denotes the abundance of haplotype 1, the most abundant haplotype overall. Private haplotypes report haplotypes detected only in one site, whereas non‐private haplotypes describe haplotypes (other than H1) found in more than one site. Numbers in brackets denote the abundance of given haplotype in a site.

**TABLE 2:** Summary of intrasample microsatellite variation, including sample size (*S*), the mean number of alleles (*N*_*A*_), total number of private alleles (*N*_*P*_), mean allelic richness (*A*_*R*_), observed (*H*_*O*_) and expected heterozygosities (*H*_*E*_) and *F*_IS_ values (significant values denoted by *) reported for European smelt *Osmerus eperlanus*.

Sample	*S*	*N*_*A*_	*N*_*P*_	*A*_*R*_	*H*_*O*_	*H*_*E*_	*F*_IS_
Cree	120	5.3	2	3	0.459	0.475	0.038
Tamar	41	2.9	0	2.2	0.261	0.279	0.078*
Thames	102	5.6	1	2.6	0.275	0.307	0.107*
Wash	105	5.5	0	2.5	0.301	0.297	−0.008
Forth	118	6.2	4	2.7	0.317	0.342	0.074*
Scheldt	35	5.3	0	2.9	0.328	0.397	0.190*
Waddenzee	125	9.3	4	3.5	0.414	0.434	0.052*
Weser	108	7.5	3	3.0	0.312	0.327	0.054*
Eider	109	8.2	1	3	0.307	0.339	0.097*
Curonian Lagoon	111	9.3	8	3.7	0.453	0.481	0.062*
Gulf of Riga	91	8.7	3	3.7	0.442	0.444	0.01
Lake Peipsi	17	4.4	0	3.2	0.41	0.453	0.119*

### 2.2 Ethical statement

The collection of samples complied with local regulations and permits.

### 2.3 Analysis of genetic data

mtDNA sequences were edited and aligned using BIOEDIT (Hall, 1999), and all subsequent analyses were performed using ARLEQUIN 3.1 (Excoffier & Lischer, 2010) unless stated otherwise. Genetic variation was described using indices of haplotype and nucleotide diversity (*h* and π, respectively). A minimum spanning network was constructed using NETWORK (www.fluxus-engineering.com/sharenet.htm). Differentiation between pairs of samples was quantified using pair‐wise Φ_ST_ with significances assessed by 10,000 permutations and following Bonferroni's correction (Rice, 1989). Fu's *Fs* (Fu, 1997) and Tajima's D (Tajima, 1989) tests were used to test for deviations from mutation‐drift equilibrium that could be attributed to selection and/or population size changes. Mismatch distributions, the frequency distributions of pair‐wise differences between haplotypes within a sample and simulated distributions under a model of demographic expansion were compared using the sum of squared deviations (SSD) as a test statistic, with significance assessed after 10,000 bootstrap replications. The timing of expansions (*T*) was estimated using the formula *T* = τ/2*u* (Rogers & Harpending, 1992), assuming a mutation rate (*u*) of 1.0 or 1.7% considered typical for marine fish and applied to osmerids (Skurikhina et al., 2018).

For microsatellite data, genetic variation was characterised for each locus and within samples using the number of alleles and private alleles (*N*_*A*_ and *N*_*P*_, respectively), allele richness (*A*_*R*_), observed and expected heterozygosities (*H*_*O*_ and *H*_*E*_, respectively) and inbreeding coefficient (*F*_IS_), calculated using GENALEX 6.2 (Peakall & Smouse, 2006) and FSTAT (Goudet, 1995). Genotype‐frequency conformance to Hardy–Weinberg equilibrium (HWE) expectations and genotypic linkage disequilibrium between loci were tested using exact tests (10,000 batches, 5000 iterations) in GENEPOP 3.3 (Raymond & Rousset, 1995). Genetic differentiation between and among samples was quantified using global and pair‐wise *F*_ST_ values, with significance assessed by 10,000 permutations in FSTAT and following Bonferroni's correction (Rice, 1989). To investigate if stepwise‐like mutations have contributed to genetic differentiation, an allele size permutation test was employed using SPAGeDi version 2.1 (Hardy & Vekemans, 2002). Confidence intervals were calculated with 10,000 permutations of allele sizes among alleles within each locus. *F*_ST_ matrices were visualised using principal co‐ordinate analysis (PCoA) in GENALEX. Mantel tests implemented in GENALEX were used to test for correlation between genetic (pair‐wise *F*_ST_) and geographical (shortest sea distances) distances between sample sites [i.e., isolation by distance (IBD)]. The individual‐based Bayesian clustering method implemented in STRUCTURE (Pritchard et al., 2000) was used to identify and assign individuals to genetically differentiated groups without any prior information. Each run consisted of a burn‐in of 10^6^ steps followed by 5 × 10^6^ steps with 10 runs performed for *K* (number of putative clusters) values ranging from 1 to 12. Optimal models were assessed using the statistics developed by Puechmaille (2016) (MEDMEDK, MEDMEAK, MAXMEDK and MAXMEAK), which are considered suitable in cases where there are a hierarchical structure and uneven sample sizes. Randomisation procedures in FSTAT were used to detect significant differences in heterozygosity, *A*_*R*_, *F*_IS_, *F*_ST_ and relatedness among user‐defined groups of samples following 10,000 permutations.

To test for evidence of recent genetic bottlenecks, we first used the heterozygosity excess method in the programme BOTTLENECK 1.2.02 (Cornuet & Luikart, 1996; Piry et al., 1999). The Wilcoxon sign‐ranked test was performed using 5000 permutations for the three mutation models [infinite allele model (IMM), stepwise mutation model (SMM) and two‐phase model (TPM)]. Second, bottlenecked populations are also expected to show a characteristic ‘mode‐shift’ in the frequency distribution of alleles away from L‐shaped distribution expected under mutation‐drift equilibrium (Luikart et al., 1998). BOTTENECK was used to generate a qualitative descriptor of such mode shifts for each site.

## 3 RESULTS

### 3.1 mtDNA diversity

**FIGURE 2:**
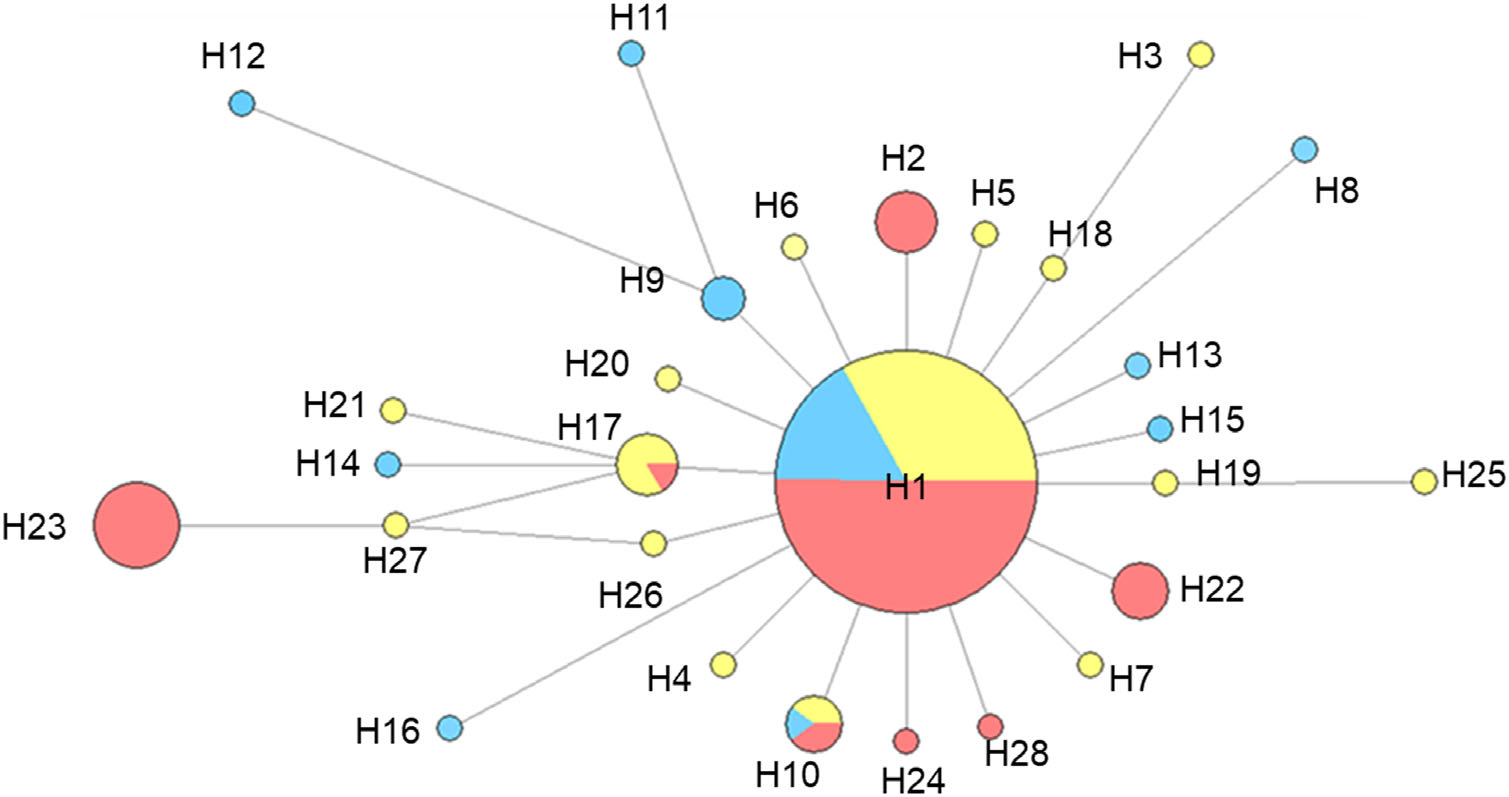
Haplotype network with disc sizes proportional to haplotype abundance in European smelt *Osmerus eperlanus*. Coloured pie slices reflect regional abundances [red = UK and Ireland; yellow = North Sea (Europe); blue = Baltic].

A 638‐bp mtDNA sequence was aligned across 184 individuals, revealing 28 haplotypes. One haplotype (haplotype 1) was proportionally dominant with an overall frequency of 0.76 and was the most abundant in all locations. Of the remaining haplotypes, only 3 were found in more than one site, 22 occurred as singletons, whereas haplotype 2 was found in multiple individuals but only in Cree smelt (Table 1). The overall haplotype diversity was 0.52 and ranged from 0 to 0.72 across locations. The haplotype genealogy conformed to a classic star‐shaped pattern with haplotype 1 at the centre (Figure 2). All haplotypes were closely related with a maximum of two site differences between adjacent haplotypes and five site differences between maximally divergent haplotypes (Figure 2). The overall nucleotide diversity was 0.001. The majority (65 of 91) of pair‐wise Φ_ST_ values were non‐significant. Significant Φ_ST_ results were largely associated with specific samples (Cree 10 of 13 pair‐wise tests significant, Thames 9 of 13 and the Wash 7 of 13, respectively) and exhibited no geographical pattern (Table S2). Across all samples, significant negative values were obtained for Tajima's D (−2.32, *p <* 0.0001) and Fu's *F*_s_ (−29.42, *p <* 0.0001). The mismatch distribution aligned with the null model of a demographic expansion [P (SSD) = 0.76] and yielded a τ‐value of 0.15 (Figure S1). Assuming a 1% mutation rate (i.e., 2% divergence rate), this resulted in an estimated time of expansion of 11,755 years before present (BP). Employing the 1.7% mutation rate, the expansion time was estimated at 6,915 years BP.

### 3.2 Microsatellite diversity

The total number of alleles per locus across all samples ranged from 6 to 27 (average = 13.7). Across all samples and loci, the mean observed and expected heterozygosities were 0.36 and 0.38, respectively (Table 2). Global tests revealed no significant linkage disequilibrium between any pair of loci. Single locus by sample HWE tests reported significant heterozygote deficits in 43 out of 144 comparisons (at critical *p* = 0.05), with significant positive multilocus *F*_IS_ values obtained for 9 out of 11 samples (Table 2). Inspection of single‐locus results indicated that heterozygote deficits were not associated with any one locus or location. For example, each locus showed heterozygote deficits at an average of three populations.

**FIGURE 3:**
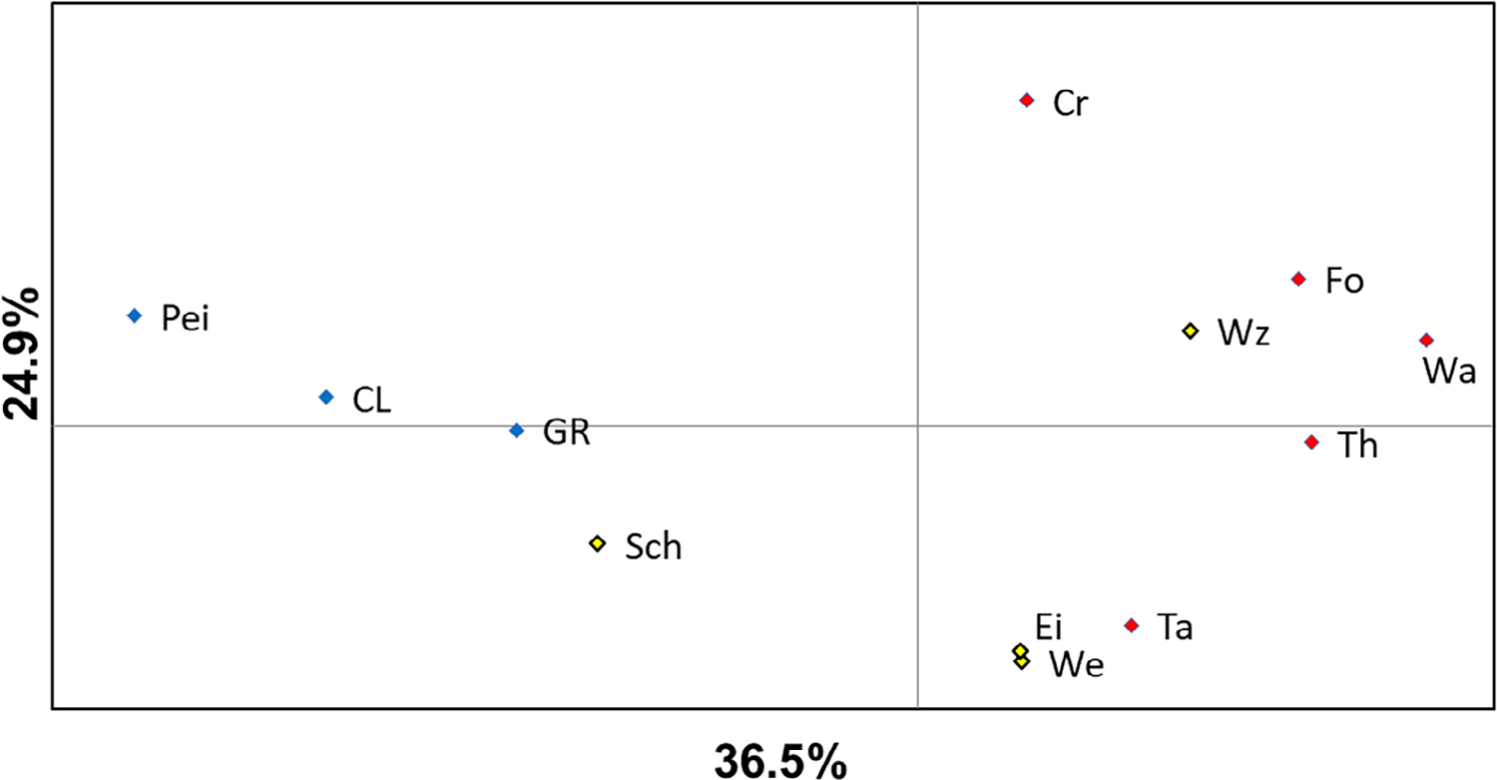
Principal co‐ordinate analysis (PCoA) of pair‐wise *F*_ST_ among European smelt *Osmerus eperlanus* samples based on 12 microsatellites. Sample names correspond to Figure 1 and are coloured based on region [UK = red; North Sea (Europe) = yellow; Baltic = blue].

**FIGURE 4:**
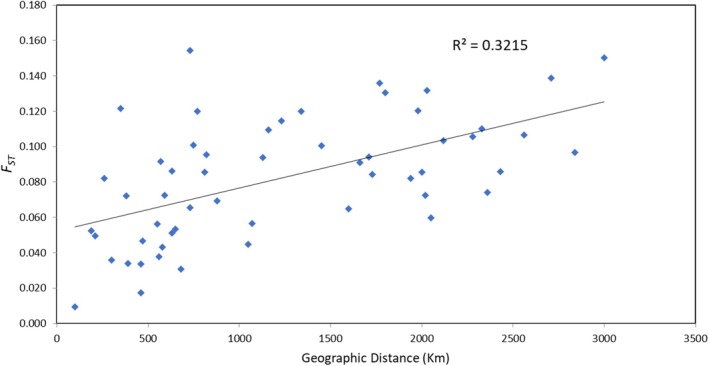
Mantel test assessing the correlation between genetic distance (*F*_ST_) and geographical distance (minimum sea distance) between pairs of anadromous samples (i.e., excluding the landlocked Lake Peipsi sample) of European smelt *Osmerus eperlanus*.

*F*_ST_ analyses revealed a high level of genetic differentiation among samples. Global *F*_ST_ was 0.15 (*p* < 0.001), and all pair‐wise *F*_ST_ values between pairs of samples were significant following Bonferroni's correction (Table 3). Allele size permutation analysis did not report *R*_ST_ to be greater than *F*_ST_. PCoA of *F*_ST_ revealed a clear separation between the Baltic and Atlantic samples (Figure 3). The Scheldt sample was more similar to the Baltic samples than the other more eastern North Sea samples (Figure 3). Among the non‐Baltic samples, the Cree also exhibited a high level of divergence from the other UK and southern North Sea sites (Figure 3). Mantel tests revealed a significant correlation between pair‐wise *F*_ST_ and minimum sea distance (*R* ^2^ = 0.321; *p* = 0.01; Figure 4). This correlation was also significant when restricted to only the non‐Baltic samples (*R* ^2^ = 0.315; *p* = 0.01).

**TABLE 3:** *F*_ST_ values between pairs of samples of European smelt *Osmerus eperlanus* based on 12 microsatellite loci.

	1	2	3	4	5	6	7	8	9	10	11
1 – Cree											
2 – Tamar	0.154										
3 –Thames	0.114	0.056									
4 – Wash	0.100	0.101	0.036								
5 – Forth	0.094	0.110	0.065	0.047							
6 – Scheldt	0.120	0.086	0.082	0.122	0.120						
7 – Waddenzee	0.065	0.069	0.034	0.034	0.031	0.072					
8 – Weser	0.136	0.045	0.051	0.092	0.095	0.038	0.052				
9 – Eider	0.131	0.056	0.053	0.073	0.086	0.043	0.050	0.009			
10 – Curonian Lagoon	0.139	0.107	0.103	0.132	0.120	0.060	0.084	0.094	0.091		
11 – Gulf of Riga	0.150	0.097	0.086	0.110	0.106	0.074	0.072	0.086	0.082	0.017	
12 – Lake Peipsi	0.146	0.151	0.147	0.187	0.173	0.083	0.128	0.138	0.142	0.045	0.076

*Note*: All values were significant at *p* < 0.001.

**FIGURE 5:**
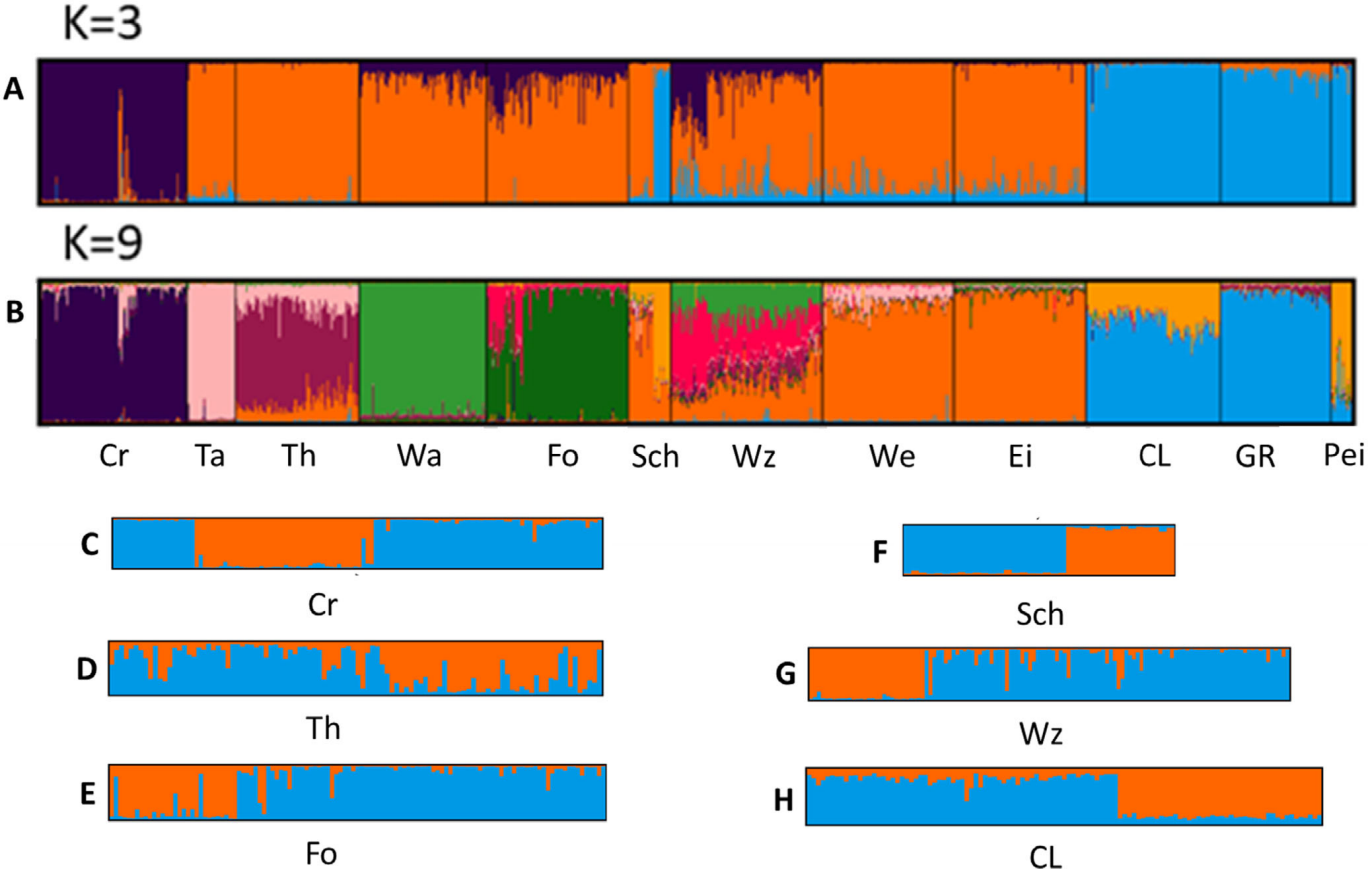
Bar plots depicting clustering patterns among samples of *Osmerus eperlanus* for models of *K* = 3 and *K* = 9 [optimal according to Puechmaille (2016)]. Also shown are bar plots for estuary samples for which *K* = 2 was found to be the optimal model (C = Cree; D = Thames; E = Forth; F = Scheldt; G = Waddenzee; H = Curonian Lagoon).

The individual‐based STRUCTURE Bayesian clustering broadly aligned with *F*_ST_ results in indicating extensive differentiation. At *K* = 3, one group comprised the Cree sample, another group included the other UK and southern North Sea samples and the third group consisted of the Baltic samples (Figure 5). Running the analysis for higher values of *K* revealed further clustering among samples, most obviously among the UK sites, which became delineated from one another and the southern North Sea sites. At *K* = 9 (which was the consensus optimal model; Figure S2), each of the five UK sites formed a discrete cluster, with structuring less apparent among the southern North Sea and Baltic regional groups (Figure 5). The degree of among‐site differentiation within these three geographical regions was evident in corresponding global *F*_ST_ values (UK = 0.154, southern North Sea = 0.081, Baltic = 0.046).

STRUCTURE was rerun for each site individually for *K* = 1–4 and reported evidence of two genetic groups within the Cree, Forth, Thames, Scheldt, Waddenzee and Curonian Lagoon (Figure 5). For each of these samples, individuals were separated based on group membership, and a within‐river *F*_ST_ calculated. These within‐river *F*_ST_ values were as follows: Cree = 0.067, Thames = 0.034, Forth = 0.07, Scheldt = 0.228, Waddenzee = 0.057, Curonian Lagoon = 0.057. Each group reported similar levels of variation in their within‐river counterpart, with within‐river groups exhibiting broadly similar levels of differentiation from other populations. A notable exception was the Scheldt for which *F*_ST_‐based analysis clustered one group with the Baltic samples, with the other group clustering with the North Sea samples, in line with the STRUCTURE analysis.

Summary indices of variation for each sample site are reported in Table 2. Out of 158 total alleles identified, 28 occurred as private alleles with an average frequency of 0.013. The Curonian Lagoon had the highest number of private alleles. There was a broad regional trend of highest levels of polymorphism (*A*_*R*_, *H*_*E*_, *H*_*O*_) for the Baltic sites and lowest for UK, with the southern North Sea sites being somewhat intermediate. This was also evident from group‐based randomisation tests, which revealed significantly different (*p* = 0.008) *A*_*R*_ values for the UK (*A*_*R*_ = 2.6), southern North Sea (*A*_*R*_ = 3.1) and Baltic (*A*_*R*_ = 3.6). The Cree (UK) and Waddenzee (southern North Sea) deviated from the broad regional pattern and exhibited levels of genetic variation generally higher than other non‐Baltic sites. All samples reported non‐significant results for the heterozygote excess bottleneck tests and no evidence of mode shifts. This was also the case for analyses performed for the partitioned within‐river groups.

## 4 DISCUSSION

The aim of our study was to investigate genetic structure in European smelt across a large part of its range in response to concerns about recent declines. mtDNA revealed a shallow phylogeographic structure, comprising a star‐like genealogy with no deep genetic breaks and low nucleotide and haplotype diversity overall. In contrast, microsatellites revealed pronounced spatial coherent genetic structuring among samples, including a significant correlation between *F*_ST_ and geographical distance and separation between Baltic and Atlantic samples. At the regional level, the UK exhibited the strongest genetic differentiation among, and lowest genetic variation within, sites. In addition to differentiation among rivers and lakes, individual clustering analyses revealed evidence of substructuring within some river systems. Across all samples, the species exhibited low levels of both mtDNA and microsatellite variation.

### 4.1 Broadscale structuring and potential role(s) for pre‐ and postglacial divergence

Phylogeographic studies of European freshwater fish have frequently revealed the existence of distinct lineages that diverged in allopatric glacial refugia (e.g., Mäkinen & Merilä, 2008; McKeown et al., 2010; Nesbo et al., 1999; Volckaert et al., 2002). The occurrence of only a single lineage in smelt represents a less common pattern that is similar to that reported for Arctic charr *Salvelinus alpinus* (Brunner et al., 2001), catfish *Silurus glanis* (Triantafyllidis et al., 2002) and weather loach *Misgurnus fossilis* (Bohlen et al., 2007) in European waters. It also differs from the phylogeographic structure reported for *O. mordax* (Bradbury et al., 2011) and *Osmerus dentex* (Skurikhina et al., 2018), which comprise glacially diverged regional lineages in North America/Canada and Eurasia, respectively. In contrast to the shallow mtDNA structuring, microsatellites revealed an unexpectedly high level of genetic differentiation (global *F*_ST_ = 0.15). Although the phylogeographic utility of microsatellites has been questioned, they have also revealed glacial vicariance not detected by mtDNA in some cases (Finnegan et al., 2013). However, the allele size permutation test in the present study provided no support for a significant contribution of mutation that might be expected if there was some prolonged vicariance (i.e., population isolation for more than 2000 generations; Estoup & Angers, 1998; Tonteri, Titov, et al., 2005, Tonteri, Veselov, et al., 2005). Overall, the mtDNA data indicate that smelt within the studied area are derived from a cohesive ancestral population, and the extensive genetic structuring revealed by microsatellites can be largely attributed to drift and restricted gene flow in the postglacial period.

Although the overall level of nuclear structure was similar to values reported among European populations of other anadromous species, for example, Atlantic salmon *Salmo salar* (King et al., 2001; Tonteri, Titov, et al., 2005, Tonteri, Veselov, et al., 2005; Säisä et al., 2005; Finnegan et al., 2013), they were well above values reported for other osmerids. For example, the overall *F*_ST_ was an order of magnitude higher than that reported for anadromous *O. mordax* across its entire northwest Atlantic range in the United States (mean *F*_ST_ = 0.015, range 0–0.08; Kovach et al., 2013) and mainland Canada (mean *F*_ST_ = 0.017 across New Brunswick and Nova Scotia; Bradbury, Coulson, et al., 2006). The level of structuring reported here was comparable to those considered anomalously high among Newfoundland smelt (*F*_ST_ = 0.11; Bradbury et al., 2008b). All pair‐wise *F*_ST_ values were statistically significant, and there was a significant correlation between genetic and geographical distances among sites (i.e., IBD) at various spatial scales. This IBD signature indicates that temporal and/or sampling (e.g., family) effects have not confounded spatial patterns (Waples, 1998). IBD patterns have been widely reported among anadromous salmonids (Fraser et al., 2011), whitefish (Harris & Taylor, 2010) and *O. mordax* (Bradbury et al., 2008b; Kovach et al., 2013) and linked to combinations of restricted dispersal and behaviours such as homing. Although there is some evidence of limited adult dispersal for *O. eperlanus* (Elliott et al., 2023; Tulp et al., 2013), dispersal at the various adult, larval and juvenile stages has been extensively studied in *O*. *mordax* for which combined genetic and otolith microchemistry analyses have revealed limited dispersal of larvae and adults from natal estuarine habitats/areas (Bradbury et al., 2008a, 2008b). Demonstrated ontogenetic shifts in vertical migration behaviour of larvae have been implicated as playing a role in localised larval retention (Bradbury, Gardiner, et al., 2006; Ouellet & Dodson, 1985), with otolith data suggesting that larvae and juveniles may not reach marine habitats (Bradbury et al., 2008a, 2008c). Bradbury et al. (2008c) reported mixing of juveniles from genetically differentiated spawning groups and suggested that the maintenance of genetic differentiation in such cases could reflect a combination of natal homing and/or selection against immigrants prior to reproduction. Local adaptation to variations in environmental conditions may also be a prominent factor in driving the strong divergence between the Atlantic and Baltic samples, and further sampling of populations between the Eider (North Sea) and Curonian Lagoon (Baltic) may reveal barriers to gene flow associated with the North Sea–Baltic transition rather than a clinal pattern, as seen in other fish (Johannesson & André, 2006; McKeown et al., 2020).

### 4.2 Pronounced population genetic structure among UK sites

*F*_ST_ and STRUCTURE results both revealed a higher level of differentiation among the UK samples in comparison to the other regions (i.e., continental Europe and Baltic). Genetic structure is shaped by the antagonistic interactions between gene flow and genetic drift. Although intrapopulation genetic diversity provided evidence of higher levels of genetic drift for UK populations (discussed later), the overall differentiation among UK sites still retained an IBD signal, implicating a role for restricted gene flow. The decline in smelt populations has arguably been most extreme in the UK. Of the 59 rivers or estuaries supporting anadromous smelt populations in England and Wales, many are greatly reduced or have disappeared altogether (Graham et al., 2021; Maitland, 2003). Considering the suspected limited ontogenetic dispersal distances for smelt, estimated to be ~1.5 km per generation for *O. mordax* (Bradbury et al., 2008b), the overall higher genetic differentiation among UK sites could be linked to loss or absence of stepping‐stone populations within a broader IBD system. In this context, it is noteworthy that the Cree sample showed a strong level of differentiation. Within the Irish Sea coastal area between the Cree and Tamar sites, there are only three extant Welsh populations of smelt recorded (Dee, Conwy and Nevern that was ‘discovered’ in 2013; Graham et al., 2021), reflecting a pronounced absence of stepping‐stone populations in this area. This absence of stepping‐stone populations is presumably due to the absence of suitable spawning areas above the head of the tide in many of the estuary/river systems along the west coast of Wales and southwest England. Furthermore, IBD does not work alone; other ecological and environmental factors can moderate the effect of distance leading to different patterns on different scales (Riginos et al., 2011). Bekkevold et al. (2020) reported a North–South differentiation for *Salmo trutta* along the eastern UK coast, which aligns geographically with the separation of the Forth from the Wash and Thames populations in the current study. The authors linked this to different environmental features, with the northern populations subjected to stronger water gradients and higher flows. The more complex topography and hydrography of the UK coastline compared to the continental Europe and Baltic sites may also drive genetic differentiation by enhancing larval retention and reducing larval reception, as has been reported to explain regional differences in genetic structure in *O. mordax* (Kovach et al., 2013) and brown carb (*Cancer Pagurus*) (McKeown et al., 2017). Finer‐scale seascape analyses will be needed to disentangle the drivers of connectivity restrictions, but the results support reduced connectivity among the sampled UK sites compared to other more open regions and indicate that a single geographic distance is unlikely to be a useful marker of connectivity throughout the species' range.

### 4.3 Within‐river structure

In addition to the genetic differentiation among rivers, clustering analysis revealed evidence of further substructure within rivers. STRUCTURE supported models of *K* = 2 for the Cree, Forth, Thames, Scheldt, Waddenzee and Curonian Lagoon samples, with the majority of individuals being robustly assigned to one of two clusters in each case. Although the limited sample information constrains our interpretation of the drivers of this structure, it is noteworthy that catchments revealing substructure here were among the largest sampled offering potential for various physical and/or ecological structuring of populations, as widely reported among salmonids (Estoup et al., 1998; McKeown et al., 2010) due to the following: (i) anthropogenic barriers (e.g., weirs) (Griffiths et al., 2009), (ii) homing to different tributary natal rivers (Stabell, 1984), (iii) patchy distribution of spawning habitat (Neville et al., 2006) or (iv) the presence of estuarine (i.e., brackish water) and in‐river (i.e., freshwater) spawners (Shpilev et al., 2005) or other ecotypic divergence (Fernández‐Alías et al., 2022). In the case of the Curonian Lagoon, the two populations described here (between‐group *F*_ST_ = 0.057) may correspond to those reported by Fernández‐Alías et al. (2022), which exhibited a similar level of differentiation (between‐group *F*_ST_ = 0.088) and were found to be intermingled within the Curonian Lagoon and adjacent Baltic waters. The highest level of within‐river divergence was reported for the Scheldt (between‐group *F*_ST_ = 0.228), with one of the groups clustering strongly with the Baltic samples. Considering the clear Atlantic–Baltic differentiation confirming restricted gene flow between these regions, the most parsimonious explanations for this are either the natural migration or artificial translocation of Baltic‐like individuals into the Scheldt and subsequent restricted interbreeding with a native/Atlantic‐like population through spatial separation in location of spawning grounds. Although the Scheldt has a long history of salmonid stocking, receiving stocked fish from diverse and often unknown origins (Houdt et al., 2005), there is no record of smelt restocking. Evidence of illegal translocation of smelt in other areas has been detected using genetic markers (Hagenglund et al., 2015), but we consider this unlikely in the Scheldt. A more likely explanation may be the natural in‐migration of smelt of Baltic origin into the Scheldt. Breine and van den Bergh (2019) report that smelt declined in abundance in the early‐20th century, with the last fish caught in 1923 before reappearing in catches in the 1990s. It is possible that native Scheldt smelt persisted at very low abundance, and that smelt of Baltic origin have entered the Scheldt and have contributed to the species' recovery since the 1990s, with both groups utilising different spawning grounds within the extensive tidal freshwater area within the river (Buysse et al., 2008). Excluding the Scheldt, each within‐river pair of populations reported similar levels of variability and estimates of differentiation to other samples, providing no evidence that one group was more isolated and/or exhibited markedly lower levels of variation. These results support and extend on those of Fernández‐Alías et al. (2022) in indicating that (i) the coexistence of demographically distinct subpopulations within catchments may be widespread and (ii) within‐river structure may be as strong, if not stronger than between‐river structure, in some cases.

### 4.4 Comparison between accessible and landlocked populations

Though this study was focused on anadromous smelt, the species is represented by both anadromous and landlocked populations. In general, comparisons between such groups in other species have typically revealed stronger interpopulation divergence and lower genetic variation among freshwater/landlocked populations (Tonteri et al., 2007; Ward et al., 1994). The strong genetic differentiation reported for the Lake Peipsi site, the only landlocked population included here, is compatible with the expectation of strong differentiation among landlocked populations. Also, in line with this is the reporting by Hagenlund et al. (2015) of an average pair‐wise *F*_ST_ of 0.15 among Scandinavian landlocked populations, representing a similar level of genetic differentiation to that reported here but over a much smaller area. However, comparison with Hagenlund et al. (2015) provides no evidence of reduced variability within the landlocked population compared to the anadromous populations studied here; rather indices of variation (e.g., *H*_*E*_ and *H*_*O*_) were slightly higher for Hagenlund et al. (2015). As the study by Hagenlund et al. (2015) analysed 15 microsatellite loci, 11 of which were analysed here, the data are readily comparable. Collectively, the patterns indicate that although life history (e.g., anadromous or landlocked) may be a good predictor of between‐population divergence, it may be less informative for predicting intrapopulation variability, which may be more dependent on regional and/or local demographic factors than life history per se.

### 4.5 Low levels of genetic variation

Levels of mtDNA variability (overall *H* = 0.52) were considerably lower than those reported for *O. dentex* across different Eurasian regions (*H* = 0.653–0.962; Skurikhina et al., 2018). This lower level of mtDNA variability for smelt compared to *O. mordax* was previously suggested by Kovpak et al. (2011). Similarly, the levels of microsatellite variation reported here, and in the studies by Hagenlund et al. (2015) and Fernandez‐Alias et al. (2022), support low levels of nuclear variation in *O. eperlanu*s, that are generally below levels reported for *O. mordax* in Canada and the United States (He = 0.58–0.87; Coulson et al., 2006; Bradbury et al., 2011; Curry et al., 2004) and the Arctic (Semenova et al., 2019). The overall levels of variability reveal *O. eperlanus* to be genetically impoverished compared to other osmerids.

What could explain the overall low levels of genetic variation in smelt? Dramatic population size reductions linked to cycles of habitat loss and release during the Pleistocene glaciations have reduced genetic variation in formerly glaciated regions for many fish species (Bernatchez & Wilson, 1998; Kontula & Väinölä, 2001), and the mtDNA (star‐like) phylogeny, neutrality tests, mismatch distributions and estimated time of expansions (following reduction) reported here are consistent with similar demographic events for smelt. Genetic variation may also be shaped by decreasing variation along postglacial colonisation routes owing to founder effects (Hewitt, 1999). Here, the Curonian Lagoon sample reported the highest number of private microsatellite alleles, with the Baltic samples in general having higher levels of genetic variation than the Atlantic samples. This contrasts with the more widely reported pattern of lower genetic variability among Baltic populations owing to historical founder effects (Johannesson & Andre, 2006). This might indicate that the species may have persisted within an eastern European/Baltic refuge as reported for other taxa (e.g., Nesbo et al., 1999; Tonteri, Titov, et al., 2005, Tonteri, Veselov, et al., 2005). Extending this idea, the lower variation among the west (UK) samples could reflect founder effects as part of colonisation from an eastern refuge. However, an important consideration is that historical patterns may be obscured by postglacial loss of genetic variation. Although the bottleneck tests reported no support for recent bottlenecks, these tests often exhibit high type II error (Le Page et al., 2000; Queney et al., 2000). The presence of haplotype 10 in both the Shannon and Gulf of Finland samples indicates that many of the low‐frequency satellite haplotypes were once more widespread and have seemingly become rarer post‐colonisation, supporting postglacial erosion of genetic variation. The high levels of variation observed in the Cree (UK) and Waddenzee (southern North Sea), compared to surrounding samples, also provide evidence of post‐colonisation genetic erosion. Finally, the low level of genetic variation might relate to life‐history characteristics. The pronounced genetic structure reported here includes within‐river substructuring, which indicates a tendency for limited effective dispersal from spawning sites. The consequent population isolation may predispose the species to low long‐term effective population size and low intrapopulation diversity, as reported for grayling (Koskinen et al., 2002).

### 4.6 Management implications

This study aimed to provide genetic information to assist conservation and management. We report an unanticipated strong level of population genetic structure that indicates that many estuaries are essentially demographically isolated and should be recognised as separate management units. There is further structuring within some estuaries emphasising the need to identify and preserve multiple spawning and nursery areas within river systems as recommended for salmonids (Griffiths et al., 2009; Primmer et al., 2006). Analysis of more recent samples and more granular sampling, including information as to the life‐history stage (i.e., juvenile, adult, spawning), will provide insight into the temporal stability of these patterns and improve understanding of the mechanisms shaping this structure (e.g., homing, extinction‐recolonisation dynamics). Moving beyond local populations, there should be a focus on improving the availability of stepping‐stone populations between estuaries through habitat restoration and protection programmes. Supplemental stocking may play a key role in both intra‐ and inter‐river efforts but should be preceded by a careful analysis of the risks relative to benefits, as well as matching stocking material to native environments. The urgency of such efforts is highlighted by the signatures of genetic erosion, particularly apparent among UK populations, which mean many populations may be poised to enter the extinction vortex (Gilpin & Soulé, 1986) or be compromised in terms of adaptive responses to environmental change.

## AUTHOR CONTRIBUTIONS

The study was designed by Ian D. McCarthy. Genetic data were collected by Alix N. Taylor, Amy J.E. Healey, Niklas Tysklind, and Niall J. McKeown. Niall J. McKeown and Ian D. McCarthy led the interpretation and drafting of the manuscript. All authors contributed to the final manuscript.

## FUNDING INFORMATION

This work was supported by a Bangor University PhD scholarship awarded to Alix N. Taylor, and an RC‐UK fellowship in fisheries genetics and conservation awarded to Martin I. Taylor. In addition, support to Niklas Tysklind was provided by CEBA: ANR‐10‐LABX‐25‐01. Niall J. McKeown also acknowledges support from the GENSPEL project funded by the Welsh Government.

## Supporting information


**Data S1.** Supporting information.

## Data Availability

All sequence data will be available on GenBank prior to publication. All microsatellite genotypes can be openly accessed on Aberystwyth University's Pure system Aberystwyth Research Portal.
