# Supplementary material for: Low genetic variation and strong genetic structure across a range of geographical scales in European smelt ( Osmerus eperlanus L.)
Source: J Fish Biol. 2025 May 29;107(3):918–31. doi: 10.1111/jfb.70093 (PMC12463765; doi:10.1111/jfb.70093)
Supplement: Supplementary file 1 — Data S1. Supporting information. [file JFB-107-918-s001.docx]

**Supplementary Table 1.** A summary of the populations of European smelt *Osmerus eperlanus* sampled for the population genetics study with the sample sizes used for the microsatellite and mtDNA analysis. DNA was extracted from muscle or scale samples of adult fish or whole larvae* except where extracted DNA samples were provided directly for analysis**.

| Sample | Country | Year sampled | Tissue  sampled | Microsatellite  N | mtDNA  N |
| --- | --- | --- | --- | --- | --- |
| Cree | Scotland | 2004 | Muscle | 120 | 16 |
| Forth^1^ | Scotland | 2003/04 | Muscle | 118 | 14 |
| The Wash^2^ | England | 2006 | Muscle | 105 | 16 |
| Thames^3^ | England | 2006 | Muscle | 102 | 17 |
| Tamar | England | 1990s | Larva* | 41 | 5 |
| Dee | Wales | 2004 | Scale | - | 3 |
| Conwy | Wales | 2004 | Scale | - | 4 |
| Shannon | Ireland | 2006 | DNA** | - | 17 |
| Scheldt | Belgium | 2006 | Muscle | 35 | 13 |
| Waddenzee | Netherlands | 2006 | Muscle | 125 | 16 |
| Weser | Germany | 2006 | Muscle | 108 | 16 |
| Eider | Germany | 2006 | Muscle | 109 | 16 |
| Curonian Lagoon | Lithuania | 2007 | Muscle | 111 | 16 |
| Lake Peipsi | Estonia | 2007 | DNA** | 17 | - |
| Gulf of Riga | Estonia | 2008 | Muscle | 91 | - |
| Gulf of Finland^4^ | Finland | 2008 | Muscle | - | 16 |

1 - samples collected at Longannet Power Station and Upper Forth Estuary

2 - samples collected on King's Lynn side of Inner wash

3 - samples collected at Canvey Island

4 - samples landed in Taamisaari

Supplementary Table 2: Pairwise ϕ_ST_ values between samples based on mtDNA sequence data with significant values following Bonferroni correction in bold (all P < 0.001).

|  | Shannon | Cree | Conwy | Dee | Tamar | Thames | Forth | Wash | Eider | Weser | Scheldt | Waddenzee | G. of Finland |
| --- | --- | --- | --- | --- | --- | --- | --- | --- | --- | --- | --- | --- | --- |
| Shannon | - |  |  |  |  |  |  |  |  |  |  |  |  |
| Cree | **0.242** | - |  |  |  |  |  |  |  |  |  |  |  |
| Conwy | -0.103 | 0.153 | - |  |  |  |  |  |  |  |  |  |  |
| Dee | -0.161 | 0.108 | 0.000 | - |  |  |  |  |  |  |  |  |  |
| Tamar | -0.070 | 0.184 | 0.000 | 0.000 | - |  |  |  |  |  |  |  |  |
| Thames | **0.246** | **0.301** | 0.114 | 0.067 | 0.145 | - |  |  |  |  |  |  |  |
| Forth | 0.027 | **0.314** | 0.000 | 0.000 | 0.000 | **0.273** | - |  |  |  |  |  |  |
| Wash | 0.190 | **0.256** | 0.058 | 0.008 | 0.090 | -0.052 | **0.214** | - |  |  |  |  |  |
| Eider | 0.015 | **0.167** | -0.135 | -0.194 | -0.101 | **0.227** | -0.009 | **0.176** | - |  |  |  |  |
| Weser | -0.025 | **0.178** | -0.117 | -0.176 | -0.084 | **0.187** | 0.012 | **0.138** | 0.001 | - |  |  |  |
| Scheldt | -0.032 | **0.204** | -0.097 | -0.156 | -0.062 | **0.173** | 0.049 | 0.125 | 0.017 | -0.044 | - |  |  |
| Waddenzee | 0.004 | **0.181** | -0.087 | -0.144 | -0.054 | **0.125** | 0.046 | 0.084 | 0.034 | -0.027 | -0.033 | - |  |
| G of Finland | 0.027 | **0.160** | -0.100 | -0.158 | -0.066 | **0.218** | 0.034 | **0.171** | 0.027 | 0.027 | 0.028 | 0.046 | - |
| Curonian Lagoon | 0.014 | **0.152** | -0.122 | -0.180 | -0.088 | **0.195** | 0.007 | **0.148** | 0.010 | 0.001 | -0.001 | 0.017 | -0.013 |


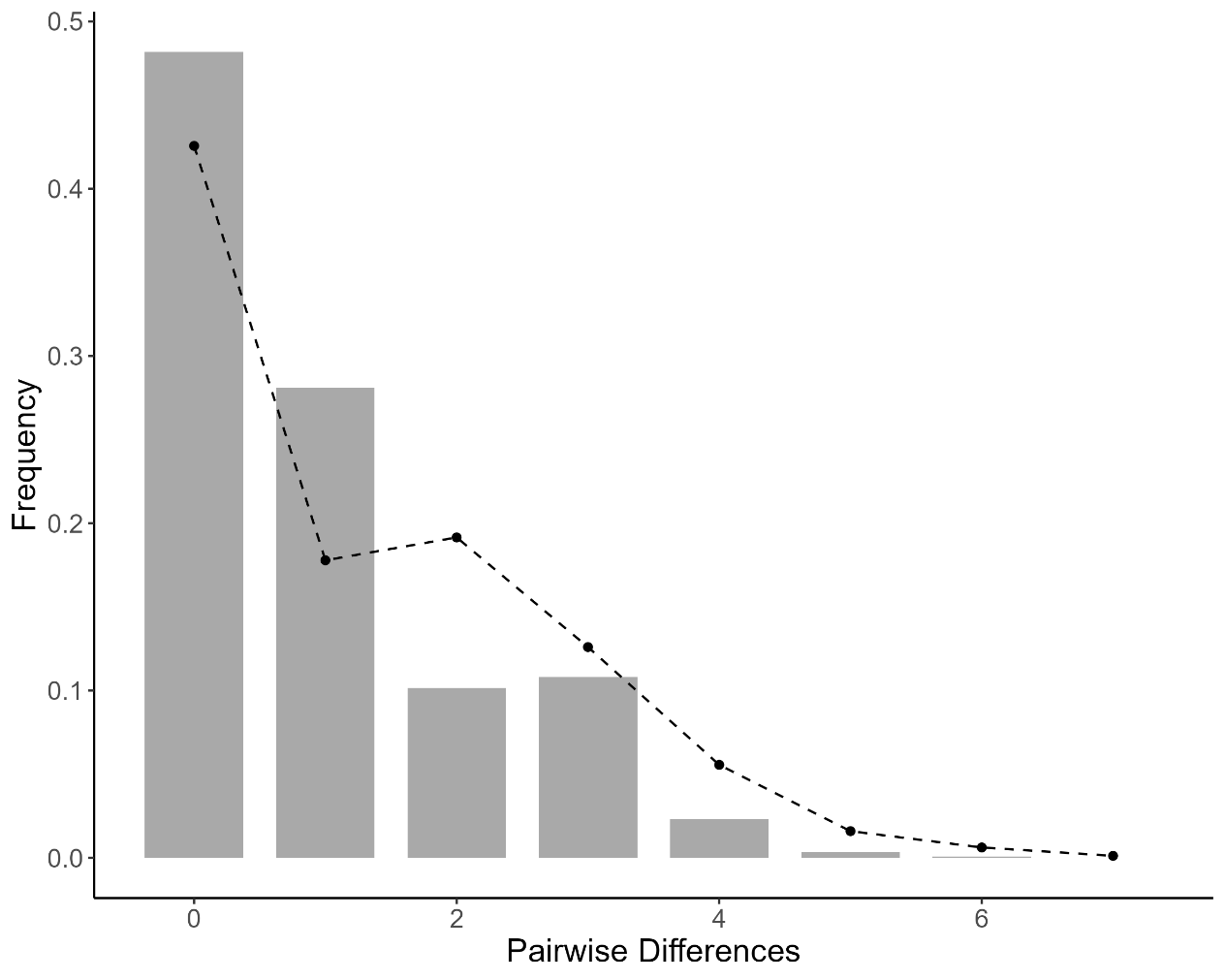


Supplementary Figure 1. Mismatch distribution histogram across all samples analysed for mtDNA variation (see Supp Table 1).


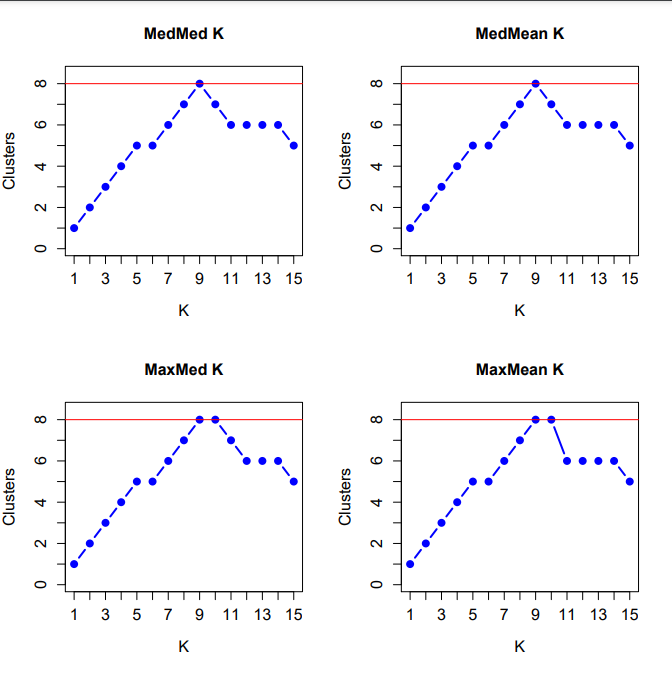


Supplementary Figure 2. Plots for the analysis of the STRUCTURE output using the 4 statistics developed by Puechmaiile (2016).
